# A diagnostic marker for superficial urothelial bladder carcinoma: lack of nuclear ATBF1 (ZFHX3) by immunohistochemistry suggests malignant progression

**DOI:** 10.1186/s12885-016-2845-5

**Published:** 2016-10-18

**Authors:** Makoto Kawaguchi, Noboru Hara, Vladimir Bilim, Hiroshi Koike, Mituko Suzuki, Tae-Sun Kim, Nan Gao, Yu Dong, Sheng Zhang, Yuji Fujinawa, Osamu Yamamoto, Hiromi Ito, Yoshihiko Tomita, Yuchi Naruse, Akira Sakamaki, Yoko Ishii, Koichi Tsuneyama, Masaaki Inoue, Johbu Itoh, Masanori Yasuda, Nobuo Sakata, Cha-Gyun Jung, Satoshi Kanazawa, Hiroyasu Akatsu, Hiroshi Minato, Takayuki Nojima, Kiyofumi Asai, Yutaka Miura

**Affiliations:** 1 Division of Diagnostic Pathology, Niigata Rosai Hospital, Japan Organization of Occupational Health and Safety, 1-7-12 Toh-un-cho, Johetsu, Niigata 942-8502 Japan; 2 Department of Molecular Neurobiology, Graduate School of Medical Sciences, Nagoya City University, 1-Chome, Kawasumi, Mizuho-cho, Mizuho-ku, Nagoya, Aichi 467-8601 Japan; 3 Division of Urology, Department of Regenerative and Transplant Medicine, Graduate School of Medical and Dental Sciences, Niigata University, 754 Ichiban-cho, Asahimachi-dohri, Cyuo-ku, Niigata, Niigata 951-8520 Japan; 4 Division of Urology, Niigata Rosai Hospital, Japan Organization of Occupational Health and Safety, 1-7-12 Toh-un-cho, Johetsu, Niigata 942-8502 Japan; 5 Noguchi Memorial Institute for Medical Research, University of Ghana, Legon, Accra, LG 581 Ghana; 6 Section of Environmental Parasitology, Faculty of Medicine, Tokyo Medical and Dental University, 1-5-45 Yushima, Bunkyo-ku, Tokyo, 113-8510 Japan; 7 Department of Oncology, Immunology and Surgery, Graduate School of Medical Sciences, Nagoya City University, 1 Kawasumi, Mizuho-cho, Mizuho-ku, Nagoya, Aichi 467-8601 Japan; 8 Laboratory of Molecular Oncology, Department of Urology, School of Medicine, Yamagata University, 2-2-2 Iida-nishi, Yamagata, Yamagata 990-9585 Japan; 9 Department of Human Science and Fundamental Nursing, School of Nursing, University of Toyama, 2630 Sugitani, Toyama, Toyama 930-0194 Japan; 10 Department of Internal Medicine, Ooshima Kurumi Hospital, 48 Kitano, Ooshima, Imizu, Toyama 939-0271 Japan; 11 Department of Pathology, Graduate School of Medicine and Pharmaceutical Sciences, University of Toyama, 2630 Sugitani, Toyama, Toyama 930-0194 Japan; 12 Department of Pathology and Laboratory Medicine, Institute of Biomedical Sciences, Graduate School of Medicine, Tokushima University, 3-18-15 Kuramoto-cho, Tokushima, Tokushima 770-8503 Japan; 13 Division of Chest Surgery, Shimonoseki City Hospital, Koyo-cho, Shimonoseki, Yamaguchi 750-8520 Japan; 14 Education and Research Support Center, School of Medicine, Tokai University, 143 Shimokasuya, Isehara, Kanagawa 259-1193 Japan; 15 Department of Diagnostic Pathology, International Medical Center, Saitama Medical University, 1397-1 Yamane, Hidaka, Saitama 350-1298 Japan; 16 Department of Biochemistry, Showa Pharmaceutical University, 3-3165 Higashi-tamagawagakuen, Machida, Tokyo 194-8543 Japan; 17 Department of Neurophysiology and Brain Science, Graduate School of Medical Sciences, Nagoya City University, 1 Kawasumi, Mizuho-cho, Mizuho-ku, Nagoya, Aichi 467-8601 Japan; 18 Department of Molecular and Cellular Biology, Graduate School of Medical Sciences, Nagoya City University, 1 Kawasumi, Mizuho-cyo, Mizuho-ku, Nagoya, Aichi 467-8601 Japan; 19 Department of Medicine for Aging in Place and Community-Based Medical Education, Graduate School of Medical Sciences, Nagoya City University, 1 Kawasumi, Mizuho-cho, Mizuho-ku, Nagoya, Aichi 467-8601 Japan; 20 Department of Pathology and Laboratory Medicine, Kanazawa Medical University, 11-1 Daigaku, Uchinada, Kahoku, Ishikawa 920-0293 Japan

**Keywords:** ATBF1, urothelial bladder carcinoma, nuclear localization signals, prognostic marker

## Abstract

**Background:**

Pathological stage and grade have limited ability to predict the outcomes of superficial urothelial bladder carcinoma at initial transurethral resection (TUR). AT-motif binding factor 1 (ATBF1) is a tumor suppressive transcription factor that is normally localized to the nucleus but has been detected in the cytoplasm in several cancers. Here, we examined the diagnostic value of the intracellular localization of ATBF1 as a marker for the identification of high risk urothelial bladder carcinoma.

**Methods:**

Seven anti-ATBF1 antibodies were generated to cover the entire ATBF1 sequence. Four human influenza hemagglutinin-derived amino acid sequence-tagged expression vectors with truncated ATBF1 cDNA were constructed to map the functional domains of nuclear localization signals (NLSs) with the consensus sequence KR[X10-12]K. A total of 117 samples from initial TUR of human bladder carcinomas were analyzed. None of the patients had received chemotherapy or radiotherapy before pathological evaluation.

**Results:**

ATBF1 nuclear localization was regulated synergistically by three NLSs on ATBF1. The cytoplasmic fragments of ATBF1 lacked NLSs. Patients were divided into two groups according to positive nuclear staining of ATBF1, and significant differences in overall survival (*P* = 0.021) and intravesical recurrence-free survival (*P* = 0.013) were detected between ATBF1+ (*n =* 110) and ATBF1− (*n =* 7) cases. Multivariate analysis revealed that ATBF1 staining was an independent prognostic factor for intravesical recurrence-free survival after adjusting for cellular grading and pathological staging (*P* = 0.008).

**Conclusions:**

Cleavage of ATBF1 leads to the cytoplasmic localization of ATBF1 fragments and downregulates nuclear ATBF1. Alterations in the subcellular localization of ATBF1 due to fragmentation of the protein are related to the malignant character of urothelial carcinoma. Pathological evaluation using anti-ATBF1 antibodies enabled the identification of highly malignant cases that had been overlooked at initial TUR. Nuclear localization of ATBF1 indicates better prognosis of urothelial carcinoma.

**Electronic supplementary material:**

The online version of this article (doi:10.1186/s12885-016-2845-5) contains supplementary material, which is available to authorized users.

## Background

Transurethral resection (TUR) is a standard initial treatment for urothelial carcinoma (UC) of the bladder. Approximately 70 % of UC of the bladder is superficial at initial diagnosis. However, approximately 60 % of patients with superficial UC experience recurrence and 10–20 % progress to invasive cancer [1]. Standard prognostic features, such as pathological stage and grade, have limited ability to predict disease outcomes [2], underscoring the need to identify accurate markers to predict the prognosis of UC.

The AT-motif binding factor 1 (ATBF1, ZFHX3) gene was originally identified as a suppressor of α-fetoprotein (AFP) gene transcription and shown to function by binding to the AT-rich element in the promoter region [3, 4]. Frequent genomic mutations of chromosome 16q23 at the *ATBF1* gene locus are found in prostate cancer [5, 6]. The loss of ATBF1 is associated with the malignant character of AFP-producing gastric cancer [7], which is characterized by loss of heterozygosity at the ATBF1 locus [8]. By contrast, ATBF1 mutations are not frequently detected in human breast cancer, in which ATBF1-A mRNA levels are regulated at the transcriptional level and not by genetic mechanisms, deletions, or mutations [9, 10]. ATBF1 is frequently detected in the cytoplasm in many types of cancer [11–13]. Loss of the nuclear localization of ATBF1 is associated with the histopathologic progression of head and neck squamous cell carcinoma [12]. However, the prognostic value of ATBF1 in patients with UC has not been investigated to date.

The aim of the present study was to examine the prognostic value of ATBF1 as a marker for predicting overall survival and recurrence-free survival in patients with UC. The prognostic value of ATBF1 was examined by histopathological staining, and the molecular mechanisms regulating the subcellular localization of ATBF1 were investigated. The expression of ATBF1 was examined in human UC tissue specimens and cell lines by generating seven anti-ATBF1 antibodies covering the entire 404-kDa ATBF1 protein to clarify the mechanism of cleavage of ATBF1 in cancer cells. Our results indicate that the anti-ATBF1 antibodies can be used to identify highly malignant cases at initial TUR.

## Results

### Mislocalization of ATBF1 in human UC cells

**Fig. 1:**
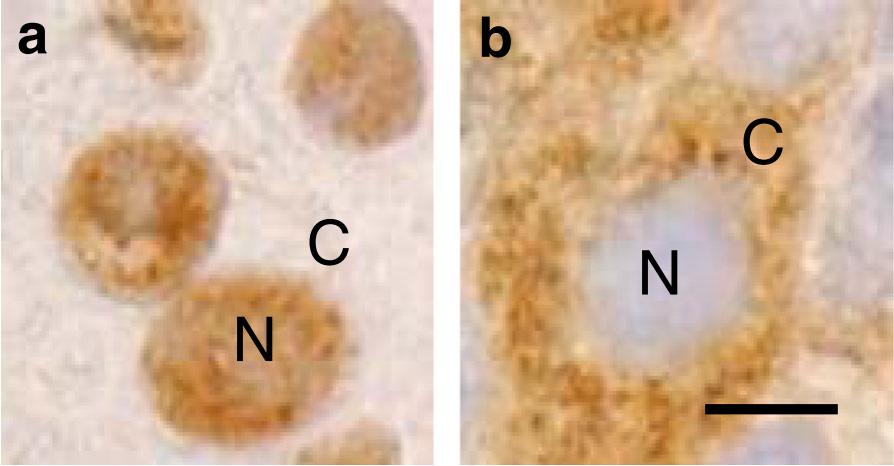
Two distinct subcellular localizations of ATBF1. **a**, UC cells expressing ATBF1 in the nucleus (N). **b**, UC cells expressing ATBF1 in the cytoplasm (C). Scale bar = 5 μm

The transcription factor ATBF1 is a DNA-binding protein that is primarily localized in the nucleus and is involved in brain development; it regulates the expression of genes involved in cell cycle arrest and cell differentiation [14, 15]. Screening of tissue sections from patients with UC by immunohistochemistry detected ATBF1 expression in the nucleus (Fig. 1a) and cytoplasm (Fig. 1b) using the same anti-ATBF1 antibody [14].

**Fig. 2:**
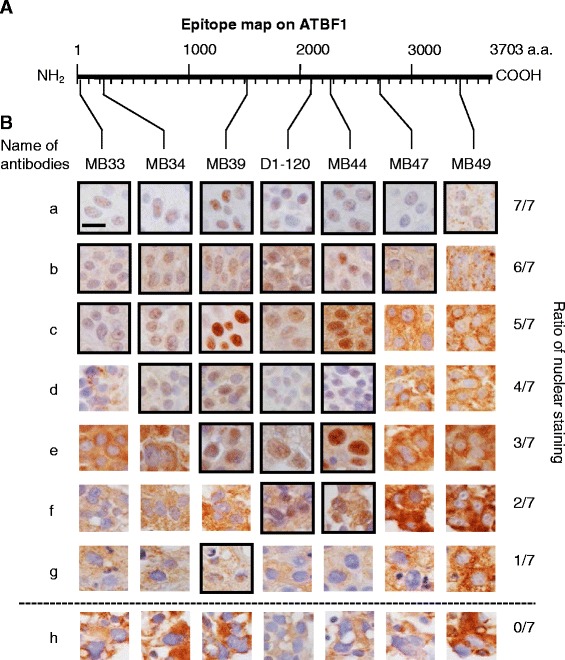
Anti-ATBF1 antibodies specifically detect distinct sections of ATBF1 localized in the nucleus or cytoplasm of UC cells. **A**, Map of the antigenic domains of the ATBF1 amino acid sequence. MB33, 16–45 aa; MB34, 238–255 aa; MB39, 1504–1520 aa; D1-120, 2114–2147 aa; MB44, 2229–2245 aa; MB47, 2759–2775 aa; MB49, 3410–3426 aa. **B**, *Left* side of the line characters a–h indicates independent cases of UC. Right side of the fraction numbers 7/7–0/7: numerators represent the number of antibodies to detect ATBF1 in the nucleus and the denominators represent the total number of antibodies (which is the fixed number 7). Scale bar = 5 μm

To determine whether the detection of ATBF1 in the cytoplasm could be attributed to the presence of ATBF1 fragments, seven specific antibodies against different ATBF1 peptides from the N-terminus to the C-terminus were generated to improve the accuracy of detection (Fig. 2A). The reactivity of these seven anti-ATBF1 antibodies against the ATBF1 protein was verified by western blot analysis in HEK293T cells expressing ATBF1 (Additional file 1: Figure S1). These antibodies detected ATBF1 in the different subcellular compartments of UC cells (Fig. 2B). The N- and C-terminal regions were frequently detected in the cytoplasm, whereas the middle sections of ATBF1 were detected in the nucleus. Based on these results, samples were divided into eight groups according to the positive nuclear staining for ATBF1, as detected by the seven independent anti-ATBF1 antibodies (Fig. 2B).

### Nuclear expression of ATBF1 predicts better clinical prognosis of human UC

**Fig. 3:**
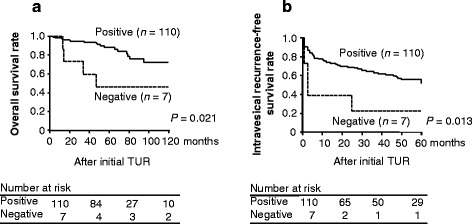
Nuclear staining of ATBF1 is a favorable indicator of patient survival. The 117 patients were divided into two groups according to ATBF1 nuclear staining (110 cases in the positive group and seven in the negative group). **a**, Overall survival according to nuclear staining of ATBF1 after 10 years by Kaplan–Meier analysis. **b**, Intravesicular recurrence-free survival analyzed under the same conditions as overall survival

The anti-ATBF1 antibodies detected positive cytoplasmic ATBF1 staining and negative nuclear staining in cancer cells, whereas normal cells expressed ATBF1 in the nucleus. To determine whether the extent of nuclear staining (Fig. 2B) was correlated with the degree of malignancy of UC, the survival of patients was analyzed by the Kaplan–Meier method. The results indicated that nuclear expression of ATBF1 was associated with better clinical prognosis of human UC regarding overall survival and intravesicular recurrence-free survival over a period of 10 years (Fig. 3).

### Nuclear ATBF1 expression is associated with pathological stage

A total of 117 primary tumors (Table 1) were screened using ATBF1 antibodies to clarify the relationship between ATBF1 and pathological stage. Spearman correlation analysis of nuclear ATBF1 staining and pathological staging in the order of low cellular grade pTa, high cellular grade pTa, pT1, and pTis showed that the correlation coefficient was −0.601, (*P* < 0.001) (Table 2). These results suggested that the subcellular localization of ATBF1 is correlated with the degree of malignancy of UC. Multivariate analyses (Tables 3 and 4) indicated that ATBF1 staining is a novel independent prognostic factor for intravesical recurrence-free survival after adjusting for cellular grade and pathological stage (*P* = 0.008), whereas it is not a significant prognostic factor for overall survival (*P* = 0.538). Of the 117 patients, 10 and 22 (Table 1) were treated with intravesical instillation of Bacille de Calmette et Guérin (BCG) and anti-cancer drugs following TUR-BT, respectively, which had no effect on overall survival (Kaplan–Meier method, Log rank test, *P* = 0.670 and 0.191, respectively). In addition, 16 patients received radical cystectomy, which had an adverse effect on overall survival (Kaplan–Meier method, Log rank test, *P* = 0.0004). Although histological grade did not differ between patients with and without radical cystectomy (chi-square test, *P* = 0.132), those who underwent radical surgery had higher T stage at TUR than patients without surgery (chi-square test, *P* = 0.019).

**Table 1:** Characteristics of patients with UC included in the study

Age at diagnosis, mean (range)	69.6 years (36–93)
Gender, male	94 cases (80.3 %)
pT stage at initial TUR	
pTa	92 cases (78.6 %)
pT1	15 cases (12.8 %)
pTis	10 cases (8.6 %)
Cellular grade at initial TUR	
Low	57 cases (48.7 %)
High	60 cases (51.3 %)
Intravesical BCG therapy	11 cases
Intravesical chemotherapy (epirubicin)	21 cases
Total cystectomy	16 cases
Bladder cancer-specific mortality	6 cases
All-cause mortality	21 cases

*BCG*, Bacille Calmette–Guérin

**Table 2:** Correlation analysis between nuclear staining of ATBF1 and pathological stage

		Pathological stage (cellular grade)			
		pTa (Low)	pTa (High)	pT1 (High)	pTis (High)
Ratio of nuclear ATBF1 staining	7/7	27	6		
	6/7	18	15	5	
	5/7	6	4	1	
	4/7	6	2	2	
	3/7		1	1	
	2/7		6	4	5
	1/7				1
	0/7		1	2	4

Spearman correlation analysis revealed a significant association (coefficient of correlation = −0.601, *P* < 0.001)

**Table 3:** Multivariate analysis of risk of overall survival

Variables		HR	95 % CI	*P*
Pathological stage	pTis	1		0.053
	pTa	0.278	0.061–1.268	
	pT1	1.292	0.319–5.238	
Cellular grade	Low	1		0.879
	High	1.104	0.309–3.935	
ATBF1 staining	Positive	1		0.538
	Negative	1.556	0.381–6.359	

*P* values by Cox’s proportional hazards model

*HR* hazard ratio, *CI* confidence interval

**Table 4:** Multivariate analysis of risk of intravesical recurrence-free survival

Variables		HR	95 % CI	*P*
Pathological stage	pTis	1		0.021*
	pTa	2.709	0.678–10.822	
	pT1	5.575	1.458–21.321	
Cellular grade	Low	1		0.713
	High	1.133	0.582–2.206	
ATBF1 staining	Positive	1		0.008*
	Negative	5.394	1.561–18.638	

*P* values by Cox’s proportional hazards model

*HR* hazard ratio, *CI* confidence interval

*Significant *P* values by Cox’s proportional hazards model

### Multiple nuclear localization signals (NLSs) drive the nuclear localization of ATBF1

**Fig. 4:**
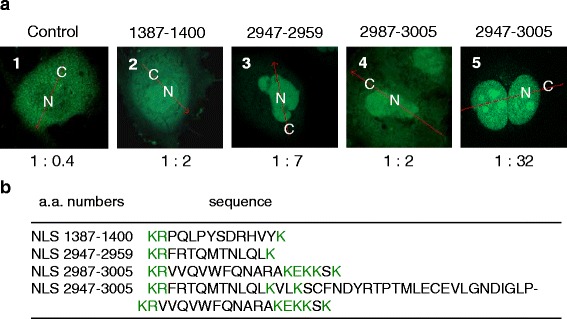
NLS synergistically drives GFP into the nucleus. Three NLSs were fused with the enhanced GFP (EGFP) gene and transfected into COS-7 cells. **a**, The numbers above the panels indicate the site of the NLSs by amino acid number on the ATBF1 protein. The ratio indicated below the panels shows the intensity of luminescence in cytoplasm (C) and nucleus (N). Panel 5 shows the synergistic effect of NLSs achieved by the tandem repeat of NLS 2947–2959 and NLS 2987–3005 to generate NLS 2947–3005. **b**, Amino acid sequences of NLS 1387–1400, NLS 2947–2959, and NLS 2987–3005, and the tandem repeated NLS 2947–3005. *Green* letters indicate the consensus sequence as an NLS

Three NLSs were identified (Fig. 4b) in addition to NLS 2615–2617 [16]. The function of each NLS (1387–1400, 2947–2959, and 2987–3005) was analyzed by generating fusion proteins with green fluorescent protein (GFP) (Fig. 4a1). Tandem repeat analysis of these two NLSs (2947–2959 and 2987–3005) showed a high concentration of GFP fusion protein in the nucleus (Fig. 4a5).

**Fig. 5:**
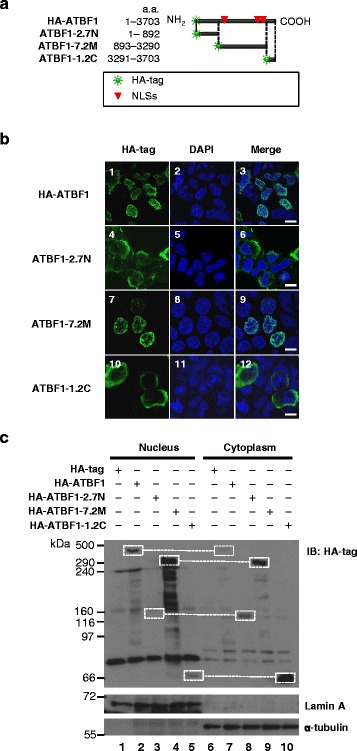
Fragments without NLS at the N- and C termini localized to the cytoplasm and fragments with NLS localized to the nucleus. **a**, Map of four HA-tagged ATBF1 expression vectors. HA-ATBF1 consisted of full-length ATBF1 cDNA. ATBF1-2.7 N consisted of 2.7-kbp of N-terminal cDNA. ATBF1-7.2 M consisted of 7.2-kbp of cDNA from the middle section encoding three NLS sequences exclusively. ATBF1-1.2C consisted of 1.2-kbp of C-terminal cDNA. The HA tag was localized at the N terminus of the ATBF1 expression vectors. The sites of the three NLSs are indicated by *red triangles*. **b**, Overexpression studies in HEK293T cells. HA-ATBF1 expressed the HA tag in the nucleus. ATBF1-2.7 N expressed the HA tag in the cytoplasm. ATBF1-7.2 M expressed the HA tag in the nucleus. ATBF1-1.2C expressed the HA tag in the cytoplasm. Scale bar = 5 μm. **c**, Full-length ATBF1 protein derived from HA-ATBF1 observed mainly in the nucleus. Truncated protein expressed by the N-terminal 2.7-kbp cDNA observed in the cytoplasmic fraction. Truncated protein expressed by the middle 7.2-kbp cDNA observed in the nuclear fraction. Truncated protein expressed by the C-terminal 1.2-kbp cDNA observed in the cytoplasmic fraction. Lamin A is a nuclear marker and α-tubulin is a cytoplasmic marker

A series of HA-tagged ATBF1 expression vectors with and without the NLSs were constructed (Fig. 5a), and the subcellular localization of these truncated ATBF1 fragments was traced by detecting the HA-tag to differentiate them from the intrinsic ATBF1 protein. Fragments lacking the NLSs at the N- and C-terminus were detected mainly in the cytoplasm (Fig. 5b, images 4, 6, 10, and 12), whereas the middle section of the ATBF1 fragment with the NLS was detected in the nucleus (Fig. 5b, images 7 and 9), similar to the full-length ATBF1 (Fig. 5b, images 1 and 3). The results of western blot analysis of HA-tagged nuclear and cytoplasmic protein fractions were consistent with those of microscopic observation. The full-length and middle parts of the ATBF1 fragment containing NLSs were mainly detected in the nuclear fraction (Fig. 5c, lanes 2 and 4). The N- and C-terminal fragments of ATBF1 were strongly detected in the cytoplasmic fraction (Fig. 5c, lanes 8 and 10).

### ATBF1 localizes to the nucleus or cytoplasm by internal cleavage to produce protein with or without NLSs

**Fig. 6:**
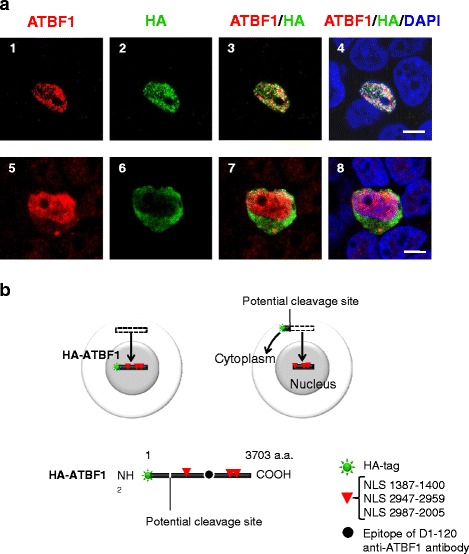
Subcellular localization of the N- and C-terminal sections of ATBF1. HEK293T cells were transfected with a HA-tagged full-length ATBF1 cDNA expression vector to observe ATBF1 under the confocal microscopy. An anti-ATBF1 antibody (D1-120) detected the middle section of ATBF1 in the nucleus (A1, A3, A4, A5, A7, A8). **a**, Most (29/30) of the cells expressed HA-tags in the nucleus with the middle part of ATBF1 (A2–A4). One cell (1/30) showed an HA-tag in the cytoplasm in contrast to ATBF1 in the nucleus (A6–A8). Scale bar = 5 μm. **b**, Schematic explaining the mechanism of abnormal localization of the N-terminal fragment of ATBF1 cleaved from the main sequence of ATBF1. The HA-tagged N-terminal fragment should be shorter than the ATBF1 protein retained in the cytoplasm. The main fragment of ATBF1 containing three NLSs (1387, 2947 and 2987) should be localized in the nucleus. The epitope of D1-120 represents the major section of ATBF1 containing three NLSs

We expected to observe full-length ATBF1 exclusively in the nucleus. Because the expression vector was designed to produce an HA-tagged ATBF1 fusion protein, the subcellular localization of the HA-tag and ATBF1 should be the same. Fragments with NLSs localized to the nucleus and fragments without NLSs localized to the cytoplasm (Fig. 5b). Both the antibody against ATBF1 and HA-tag should appear in the nucleus (Fig. 6a, images 1–4). However, anti-HA-tag antibody staining was detected in the cytoplasm (Fig. 6a, images 6–8) in addition to anti-ATBF1 antibody staining in the nucleus (Fig. 6a, images 5, 7, and 8). The ATBF1 protein must be expressed as a single molecule including the HA-tag. This paradoxical observation suggested that the protein underwent cleavage in to at least two fragments fragments with and without NLSs (Fig. 6b).

## Discussion

The transcription factor ATBF1 is mainly expressed in the nucleus, although it is frequently expressed in the cytoplasm of cancer cells [12, 13]. In the present study, we show that abnormal subcellular localization of ATBF1 in UC is derived from cleavage of ATBF1 to produce smaller fragments. Fragments of ATBF1 with NLSs localized to the nucleus, whereas fragments lacking NLSs were retained in the cytoplasm. Three NLSs (1387–1400, 2947–2959, and 2987–3005) were identified, in addition to the previously reported NLS 2615–2617 [16]. Although each NLS functions independently to drive the nuclear localization of the protein, a single NLS signal has weak activity. Tandem repeated NLSs enable efficient nuclear transportation. Because ATBF1 is large, the cooperation of multiple NLSs is needed for efficient localization to the nucleus. We found that a small N-terminal fragment of ATBF1 was released to the cytoplasm and separated from the major part of ATBF1 in the nucleus (Fig. 6). The malignant character of cancer cells may result in the cleavage of ATBF1 to produce fragments localized to the cytoplasm (Additional file 2: Figure S3).

The mechanism underlying the generation of ATBF1 fragments in cancer cells remains unclear, and the precise cleavage sites and enzymes responsible are unknown at present. This was one of the limitations of our present study. We speculate that the function of these abnormal fragments in the cytoplasm is to inhibit the function of intact ATBF1 in the nucleus. Previous studies showed that phosphatase treatment to reduce the phosphorylation of ATBF1 enhances the sensitivity to calpain and induces the degradation of ATBF1 [17]. However, there are no studies reporting increased activity of phosphatases in UC. On the contrary, alkaline phosphatase levels are low in T24 bladder cancer cells [18], which are used as the most malignant model of UC. This suggests that alkaline phosphatase levels are not the main cause of ATBF1 instability in T24 bladder cancer cells, and ATBF1 fragmentation could be mediated by an as yet unknown mechanism in T24 bladder cancer cells (Additional file 3: Figure S2).

The pathological evaluation of ATBF1 staining was consistent with the WHO classification of UC. A decrease in the nuclear localization of ATBF1 was associated with an increased degree of malignancy of UC. Pathological evaluation of ATBF1 is an effective method for detecting highly malignant cases, specifically in low-grade cases at the initial diagnosis by classical hematoxylin and eosin staining. Radical treatment of these cases at the initial stages could improve survival rates by suppressing invasion and metastasis in the long term. This hypothesis should be further evaluated in future clinical trials to test its accuracy.

The malignant characteristics of T24 bladder cancer cells require activation of signal transducer and activator of transcription (STAT)3 for epidermal growth factor induction of matrix metalloproteinase-1 and −10 (stromelysin-2). Furthermore, expression of dominant-negative STAT3 is sufficient to inhibit cell migration, invasion, and tumor formation in nude mice [19]. These results demonstrate that STAT3 phosphorylation plays a crucial role in the malignant characteristics of T24 bladder cancer cells. LIV1 (SLC39A6: solute carrier family 39 zinc transporter member 6), a breast-cancer-associated zinc transporter protein, is a downstream target of STAT3 that is essential and sufficient for the cell-autonomous role of STAT3 in the epithelial-mesenchymal transition (EMT) of zebrafish gastrula organizer cells [20]. LIV1 is essential for the nuclear localization of the zinc-finger protein Snail, a master regulator of EMT, to suppress E-cadherin. These results establish a molecular link between STAT3, LIV1, and Snail in EMT [20]. ATBF1 inhibits STAT3 signaling by interacting with protein inhibitor of activated STAT3 [21]; therefore, the fragmentation of ATBF1 may induce STAT3 hyper-activation and EMT (Additional file 4: Figure S4B).

Based on traditional histopathological examinations, patients with potentially highly malignant disease received radical cystectomy. It was evident that the adverse effects of radical surgery were not related to poorer survival outcomes. In current clinical practice, radical surgery for those with possible highly malignant disease does not improve survival compared with that in patients who are regarded as having clinocopathologically indolent disease without the need for surgery. Thus, for patients with possible malignant histopathology, we cannot provide additional usable information on traditional examinations. However, we found that the status of ATBF1 was predictive of lethal disease among those initially diagnosed with low malignant potential cancers; prompt discrimination of patients with potentially highly malignant disease using the new technology with the ATBF1 assay may lead to more efficient surgical treatment and superior prognostic outcomes.

## Conclusions

The mislocalization of ATBF1 in malignant UC cells is caused by the cleavage of the ATBF1 protein into fragments rather than the specific subcellular localization of the protein. Fragments containing a NLS localize to the nucleus, whereas those lacking a NLS are retained in the cytoplasm. Alteration of the subcellular localization of these distinct fragments of ATBF1 is related to the malignant character of UC. Pathological evaluation using anti-ATBF1 antibodies is an effective method to identify malignant cases that are overlooked at initial TUR.

## Methods

### Patients

A total of 141 patients who were admitted to the Niigata Rosai Hospital between January 1997 and October 2006 were clinically suspected of having UC of the bladder. The study focused on UC malignancy at the superficial stage for better choice of treatment and to achieve better prognosis. One hundred and thirty patients were diagnosed with superficial UC of the bladder according to the updated WHO and TNM classifications [22]. Diagnosis was made by three pathologists certified by the Japanese Society of Pathology. Two pathologists (Y.I. and K.T.) made the initial diagnosis, which was confirmed by M.K. When a clear classification was difficult, M.K. re-evaluated the evidence and made the final decision regarding classification. All samples obtained from initial TUR were formalin-fixed, paraffin-embedded blocks and were suitable for immunohistochemical analysis. Eleven patients with combined adenocarcinoma, squamous cell carcinoma, carcinosarcoma or anaplastic carcinoma were excluded. Two patients with insufficient clinicopathological data were also excluded. Follow-up details were available for 117 patients with superficial UCs. The follow-up period ranged from 1 to 120 months (median, 55 months). None of the patients received chemotherapy or radiotherapy before initial TUR.

### Cell lines

The bladder cancer cell lines RT4, HT1376, and T24 were obtained from the American Type Culture Collection (ATCC). The cells were maintained in RPMI-1640 medium supplemented with 10 % fetal bovine serum in a 37 °C, 5 % CO_2_ humidified incubator. Cell growth was measured by MTT conversion assay (Chemicon International, Temecula, CA, USA). Absorbance was evaluated in triplicate at 490 nm using a Multiplate Reader (Bio Rad, Hercules, CA, USA). COS-7 and HEK293 cells were obtained from ATCC, and cultured in Dulbecco’s modified Eagle’s medium (i; low glucose) containing 10 % fetal bovine serum without antibiotics. These cells were used for transfection of the various ATBF1 expression vectors.

### Transfection and plasmid constructs

To generate expression vectors containing the GFP gene fused with DNA fragments encoding NLSs (1387, 2947, 2987, and 2947 + 2987), four oligomer sets were prepared to produce NLSs including the fragments as follows: for NLS 1387, 4798U: gcccttcagacgcattttaat and 4906 L: tcttgaaggccaggctacac; for NLS2947, 9469U: tctgcaggcaaatctggtga and 9619 L: gtcattgcccaggacgtgac; for NLS2987, 9616U: attggactgccaaagagagt and 9741 L: tttgggtccctcataactgc; for NLS2947 + 2987, 9469U: tctgcaggcaaatctggtga and 9741 L: tttgggtccctcataactgc. These polymerase chain reaction products were subcloned into pGEM-T Easy (Promega, Madison, WI, USA) and then subcloned into the MCS (*Eco*RI-*Xba*I) site of pEGFP-C1 (Clontech, Takara Bio Inc., Otsu, Shiga, Japan). These expression vectors were transfected into COS7 cells. A series of truncated ATBF1 cDNA expression vectors was prepared from the full-length ATBF1 expression vector pHA-ATBF1 [15, 21, 23]. The truncated ATBF1 cDNA sequences were prepared by digestion with the following enzymes: *Sal*I and *Bam*HI (1–892 aa), *Bam*HI and *Sfi*I (893–3288 aa), and *Sfi*I and *Not*I (3289–3703 aa). Each fragment was subcloned into pCI-HA [15] and digested with *Sal*I and *Not*I using oligolinker-mediated ligation. These vectors were transfected into HEK 293 T cells using TransIT-293 T reagent (Mirus Bio, Madison, WI, USA) on tissue culture dishes. Cells were placed on glass slides coated with combinations of poly-L-lysine (Sigma, St Louis, MO, USA) for microscopic analysis.

### Anti-ATBF1 antibodies

The seven antibodies used in this study were rabbit polyclonal antibodies produced against polypeptides designed based on the amino acid sequence of human ATBF1. The polypeptides used were as follows: amino acid residues 16–45 (MB33), 238–255 (MB34), 1504–1520 (MB39), 2229–2245 (MB44), 2759–2775 (MB47), and 3410–3426 (MB49) (Fig. 5a). An antigen for D1-120 (MBL, Nagoya, Japan) corresponding to human ATBF1 amino acid residues 2107–2147 was produced as a glutathione-S-transferase fusion protein.

### Western blot analysis

Western blot analysis was performed on protein extracts that were separated into nuclear and cytoplasmic fractions as described by Dignam et al. [24]. Protein concentration was measured using the Bradford assay kit (Bio-Rad). Each sample was separated on a 4–20 % gradient sodium dodecyl sulfate polyacrylamide gel (Bio-Rad), and transferred to a polyvinylidene difluoride membrane (Millipore, Billerica, MA, USA). The membrane was blocked with 5 % bovine serum albumin (Fraction V; Sigma) in Tris-buffered saline containing 0.05 % Tween 20 for 1 h, and then incubated with primary antibodies for 1 h at room temperature. All antibodies were diluted in 1 % bovine serum albumin in Tris-buffered saline containing 0.05 % Tween 20. Rabbit polyclonal anti-ATBF1 antibody (antibody D1-120 in Fig. 5a; MBL, dilution 1:1000), anti-SOD antibody (Cu–Zn superoxide dismutase-1, dilution 1:1000; Stressgen, Sapphire Bioscience, Waterloo, WA, Australia), and anti-histone H3 antibody (dilution 1:150; Abcam, San Francisco, CA, USA) were used. Next, the membrane was incubated with a secondary antibody, goat anti-rabbit IgG (H + L-chain)-HRP antibody at 1:2000 (MBL) or sheep anti-mouse IgG/horseradish peroxidase (Amersham GE Healthcare, Bucks, UK, dilution 1:1000) for 1 h at room temperature, and visualized using Amersham ECL Plus (GE Healthcare) and an LAS-3000 image analyzer (Fuji Photo Film, Tokyo, Japan).

### Immunohistochemistry

The 5-μm sections of paraffin-embedded tissues and the cell blocks were deparaffinized and rehydrated by the following procedure. Antigen was retrieved with a citrated buffer (0.01 M, pH 6.0) at 110 °C for 4 min in a pressure cooker. Sections were incubated with methanol containing 0.3 % H_2_O_2_ and 1.0 % sodium azide to block endogenous peroxidase activity, and then incubated with the seven anti-ATBF1 antibodies for 1 h. Antibody dilutions were as follows: MB33 (1:50), MB34 (1:100), MB39 (1:300), D1-120 (1:300), MB44 (1:120), MB47 (1:10), and MB49 (1:5). Staining was carried out at room temperature. Immunoreactive products were detected with an anti-rabbit IgG antibody (Envision labeled polymer; Dako Cytomation, Kyoto, Japan) and visualized by diaminobenzidine chromogen. Tissue sections were counterstained with hematoxylin.

### Immunocytochemistry

Cells were fixed in 4 % paraformaldehyde in phosphate-buffered saline (PBS) at room temperature for 20 min, then washed with 0.25 % Triton-X in PBS, and blocked with 10 % normal goat serum. Cells were then incubated for 1 h at room temperature with primary antibodies against ATBF1 (D1-120; MBL), β-tubulin III (Sigma), and HA-tag (MBL). After three washes with 0.25 % Triton-X in PBS, cells were visualized using the following secondary antibodies: Alexa 488-conjugated goat anti-mouse, Alexa 546-conjugated goat anti-rabbit IgG, Alexa 488-conjugated goat anti-rat, and Alexa 546-conjugated goat anti-rat IgG (Molecular Probes, Eugene, OR, USA). Sections were incubated for 1 h in the dark, and after two washes with PBS, subjected to nuclear staining with 4′,6-diamidino-2-phenylindole (Wako, Osaka, Japan) for 5 min.

### Pathological evaluation

The immunoreactivity of ATBF1 was tested on normal human urothelium by immunohistochemistry, and on HEK293T cells with and without ATBF1 expression vector by Western blot analysis (Additional file 1: Figure S1). We used a malignant tumor lesion showing the most serious atypia in pTa and pTis. In addition, the most deeply invaded lesion in the submucosal area in pT1 was selected. In cases with multiple foci of different grades of cancer, we chose the lesion with the highest malignancy grade. In cases of pTa tumors concomitant with a pTis tumor, the pTis tumor was used to make a diagnosis. In cases of a pT1 tumor concomitant with pTa tumors, the pT1 tumor was chosen for the diagnosis.

### Statistical analysis

Spearman correlation analysis was used to examine the correlation of ATBF1 nuclear staining with pathological status of UC. The Kaplan–Meier method and the log-rank test were used to analyze differences in overall survival or recurrence-free survival between different staining groups. The association of clinicopathological parameters with the risk of overall survival and intravesical recurrence-free survival was evaluated by the Cox proportional hazards model. A value of *P* < 0.05 was regarded as significant. All analyses were performed using SPSS version 16.0 (Softonic, Chicago, IL, USA) and Prism 4.0 (GraphPad Software, La Jolla, CA, USA) on a Windows-based computer.

## Additional files

Additional file 1: Figure S1. Specificity and sensitivity of the seven anti-ATBF1 antibodies. Western blot analysis of ATBF1 in HEK293T cells using the seven anti-ATBF1 antibodies (Fig. 5a MB33, MB34, MB39, D1-120, MB44, MB47 and MB49). Lanes 1, 3, 5, 7, 9, 11, and 13 represent HEK293T cells with an HA-tag expression vector (pCI-HA). Lanes 2, 4, 6, 8, 10, 12, and 14 represent HEK293T cells containing an HA-tagged ATBF1 expression vector (pCI-HA-ATBF1). HEK293T cells were grown in DMEM supplemented with 10 % fetal bovine serum at 37 °C and 5 % CO_2_. HEK293T cells were transfected with the HA-tagged expression vector or the HA-tagged ATBF1 expression vector (HA-ATBF1) using transIT-293 reagent. (PPTX 215 kb)
Additional file 2: Figure S3. Schematic explanation of the correlation between the mislocalization of ATBF1 and the malignant characteristics of cancer cells. The transcription factor ATBF1 is a DNA-binding protein found in the nucleus that regulates the transcription of genes inducing cell cycle arrest. Cleavage of ATBF1 should impair the physiological function of ATBF1 in the nucleus as an inhibitory factor against carcinogenesis. Cleaved fragments with no NLSs indicate the malignant character of cancer cells. (PPTX 49 kb)
Additional file 3: Figure S2.
**A**, T24 cells showed the most malignant staining pattern of ATBF1 (Fig. 2B) and HT1376 showed the most benign staining pattern (Fig. 2B) similar to the staining pattern of RT4. RT4 is a cell line derived from non-malignant papilloma. Scale bar = 5 μm. **B**, Western blot analysis of D1-120 showed the loss of ATBF1 in T24 cells. The result is relevant to the pathological staining with D1-120 regarding the loss of ATBF1 in the nucleus and cytoplasm. RT-4 and HT1376 cells expressed smaller fragments of ATBF1 in the cytoplasm and larger fragments of ATBF1 in the nucleus. The results suggest that the ATBF1 in the cytoplasm was not the full length but fragments of ATBF1. **C**, T24 cells, which expressed no ATBF1 in the nucleus, grew faster than the other two cell lines expressing ATBF1 in the nucleus. (PPTX 493 kb)
Additional file 4: Figure S4. ATBF1 is highly expressed in cells at the epithelial zone associated with the expression of E-cadherin. **A**, Pathological specimens of bladder carcinoma were stained by hematoxylin and eosin, E-cadherin, and anti-ATBF1 antibodies (MB33, MB34, MB39, D10120, MB44, MB47 and MB49). Bladder lumen (L), epithelial zone (E), and mesenchymal zone (M) are shown. A transition from higher expression of ATBF1 in the E zone to lower expression of ATBF1 in the M zone was observed. The basement membrane is not clear between the E and M zones because of invasive cancer. Scale bar = 5 μm. **B**, Schematic explanation of the function of ATBF1 in inducing E-cadherin. ATBF1 interacts with protein inhibitor of activated STAT3 (PIAS3) to suppress STAT3 signaling [21]. Activation of STAT3 should activate the zinc transporter LIV1 and introduce zinc ions into the cells. The zinc ion is a regulatory factor for Snail to suppress E-cadherin. Therefore, zinc transporters play an important role in the migration of cells during embryogenesis [20]. Fragments of ATBF1 may have a dominant negative effect on suppressing STAT3, whereas they promote STAT3 signaling and suppress the expression of E-cadherin, which might be the basis of invasion and metastasis. (PPTX 1682 kb)

## Abbreviations

AFP: Alpha-fetoprotein
ATBF1: AT-motif binding factor 1
ATCC: American type culture collection
BCG: Bacille de Calmette et Guérin
DMEM: Dulbecco’s modified Eagle’s medium
EGFP: Enhanced green fluorescent protein
EMT: Epithelial-mesenchymal transition
GFP: Green fluorescent protein
HA: Hemagglutinin
kbp: Kilo base pairs
kDa: Kilodalton
LIV1 (Synonym of SLC39A6): Solute carrier family 39 (zinc transporter), member 6
MCS: Multiple cloning site
MTT: 3-(4,5-Dimethylthiazol-2-yl)-2,5-diphenyltetrazolium bromide
NLS: Nuclear localization signal
*P*: Probability
PBS: Phosphate-buffered saline
PIAS3: Protein inhibitor of activated STAT3
pT1: Tumor invades subepithelial connective tissue
pTa: Non-invasive papillary carcinoma
pTis: Carcinoma in situ
SPSS: Statistical package for the social sciences
STAT3: Signal transducer and activator of transcription 3
TNM: The TNM classification of malignant tumors
TUR: Transurethral resection
UC: Urothelial carcinoma
WHO: World Health Organization
ZFHX3: Zinc finger homeobox 3
