# Supplementary material for: A diagnostic marker for superficial urothelial bladder carcinoma: lack of nuclear ATBF1 (ZFHX3) by immunohistochemistry suggests malignant progression
Source: BMC Cancer. 2016 Oct 18;16:805. doi: 10.1186/s12885-016-2845-5 (PMC5070376; doi:10.1186/s12885-016-2845-5)
Supplement: Additional file 1: Figure S1. — Specificity and sensitivity of the seven anti-ATBF1 antibodies. Western blot analysis of ATBF1 in HEK293T cells using the seven anti-ATBF1 antibodies (Fig. 5a MB33, MB34, MB39, D1-120, MB44, MB47 and MB49). Lanes 1, 3, 5, 7, 9, 11, and 13 represent HEK293T cells with an HA-tag expression vector (pCI-HA). Lanes 2, 4, 6, 8, 10, 12, and 14 represent HEK293T cells containing an HA-tagged ATBF1 expression vector (pCI-HA-ATBF1). HEK293T cells were grown in DMEM supplemented with 10 % fetal bovine serum at 37 °C and 5 % CO2. HEK293T cells were transfected with the HA-tagged expression vector or the HA-tagged ATBF1 expression vector (HA-ATBF1) using transIT-293 reagent. (PPTX 215 kb) [file 12885_2016_2845_MOESM1_ESM.pptx]

## Slide 1
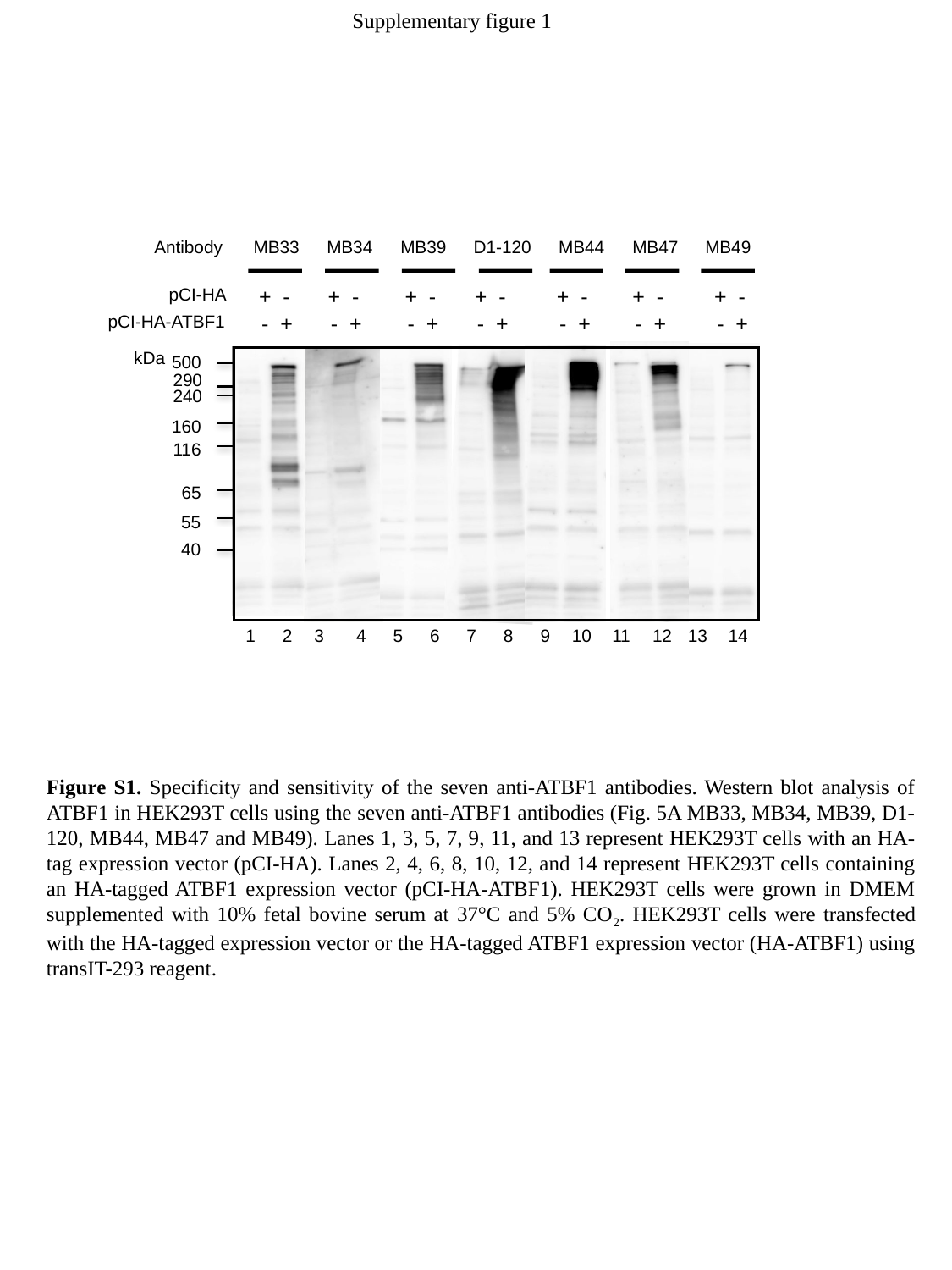

Supplementary figure 1
Antibody
MB33
MB34
MB39
D1-120
MB44
MB47
MB49
pCI-HA
+ -
- +
+ -
- +
+ -
- +
+ -
- +
+ -
- +
+ -
- +
+ -
- +
pCI-HA-ATBF1
kDa
500
290
240
160
116
65
55
40
 1
 2
 3
 4
 5
 6
 7
 8
 9
 10
 11
 12
 13
 14
Figure S1. Specificity and sensitivity of the seven anti-ATBF1 antibodies. Western blot analysis of ATBF1 in HEK293T cells using the seven anti-ATBF1 antibodies (Fig. 5A MB33, MB34, MB39, D1-120, MB44, MB47 and MB49). Lanes 1, 3, 5, 7, 9, 11, and 13 represent HEK293T cells with an HA-tag expression vector (pCI-HA). Lanes 2, 4, 6, 8, 10, 12, and 14 represent HEK293T cells containing an HA-tagged ATBF1 expression vector (pCI-HA-ATBF1). HEK293T cells were grown in DMEM supplemented with 10% fetal bovine serum at 37°C and 5% CO2. HEK293T cells were transfected with the HA-tagged expression vector or the HA-tagged ATBF1 expression vector (HA-ATBF1) using transIT-293 reagent.
