# Supplementary material for: A diagnostic marker for superficial urothelial bladder carcinoma: lack of nuclear ATBF1 (ZFHX3) by immunohistochemistry suggests malignant progression
Source: BMC Cancer. 2016 Oct 18;16:805. doi: 10.1186/s12885-016-2845-5 (PMC5070376; doi:10.1186/s12885-016-2845-5)
Supplement: Additional file 2: Figure S3. — Schematic explanation of the correlation between the mislocalization of ATBF1 and the malignant characteristics of cancer cells. The transcription factor ATBF1 is a DNA-binding protein found in the nucleus that regulates the transcription of genes inducing cell cycle arrest. Cleavage of ATBF1 should impair the physiological function of ATBF1 in the nucleus as an inhibitory factor against carcinogenesis. Cleaved fragments with no NLSs indicate the malignant character of cancer cells. (PPTX 49 kb) [file 12885_2016_2845_MOESM2_ESM.pptx]

## Slide 1
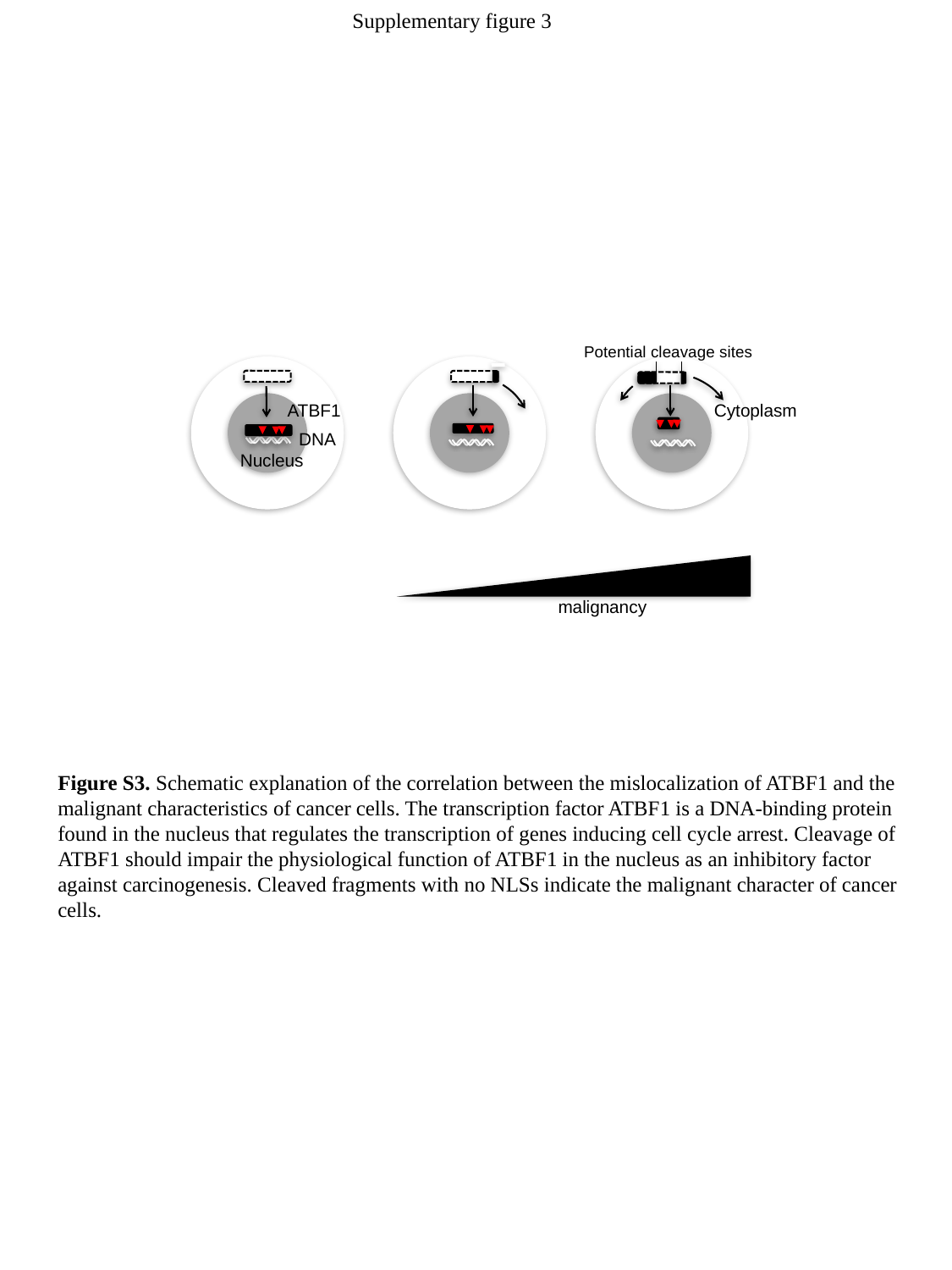

Supplementary figure 3
Potential cleavage sites
ATBF1
Cytoplasm
DNA
Nucleus
malignancy
Figure S3. Schematic explanation of the correlation between the mislocalization of ATBF1 and the malignant characteristics of cancer cells. The transcription factor ATBF1 is a DNA-binding protein found in the nucleus that regulates the transcription of genes inducing cell cycle arrest. Cleavage of ATBF1 should impair the physiological function of ATBF1 in the nucleus as an inhibitory factor against carcinogenesis. Cleaved fragments with no NLSs indicate the malignant character of cancer cells.
