# Supplementary material for: A diagnostic marker for superficial urothelial bladder carcinoma: lack of nuclear ATBF1 (ZFHX3) by immunohistochemistry suggests malignant progression
Source: BMC Cancer. 2016 Oct 18;16:805. doi: 10.1186/s12885-016-2845-5 (PMC5070376; doi:10.1186/s12885-016-2845-5)
Supplement: Additional file 3: Figure S2. — A, T24 cells showed the most malignant staining pattern of ATBF1 (Fig. 2B) and HT1376 showed the most benign staining pattern (Fig. 2B) similar to the staining pattern of RT4. RT4 is a cell line derived from non-malignant papilloma. Scale bar = 5 μm. B, Western blot analysis of D1-120 showed the loss of ATBF1 in T24 cells. The result is relevant to the pathological staining with D1-120 regarding the loss of ATBF1 in the nucleus and cytoplasm. RT-4 and HT1376 cells expressed smaller fragments of ATBF1 in the cytoplasm and larger fragments of ATBF1 in the nucleus. The results suggest that the ATBF1 in the cytoplasm was not the full length but fragments of ATBF1. C, T24 cells, which expressed no ATBF1 in the nucleus, grew faster than the other two cell lines expressing ATBF1 in the nucleus. (PPTX 493 kb) [file 12885_2016_2845_MOESM3_ESM.pptx]

## Slide 1
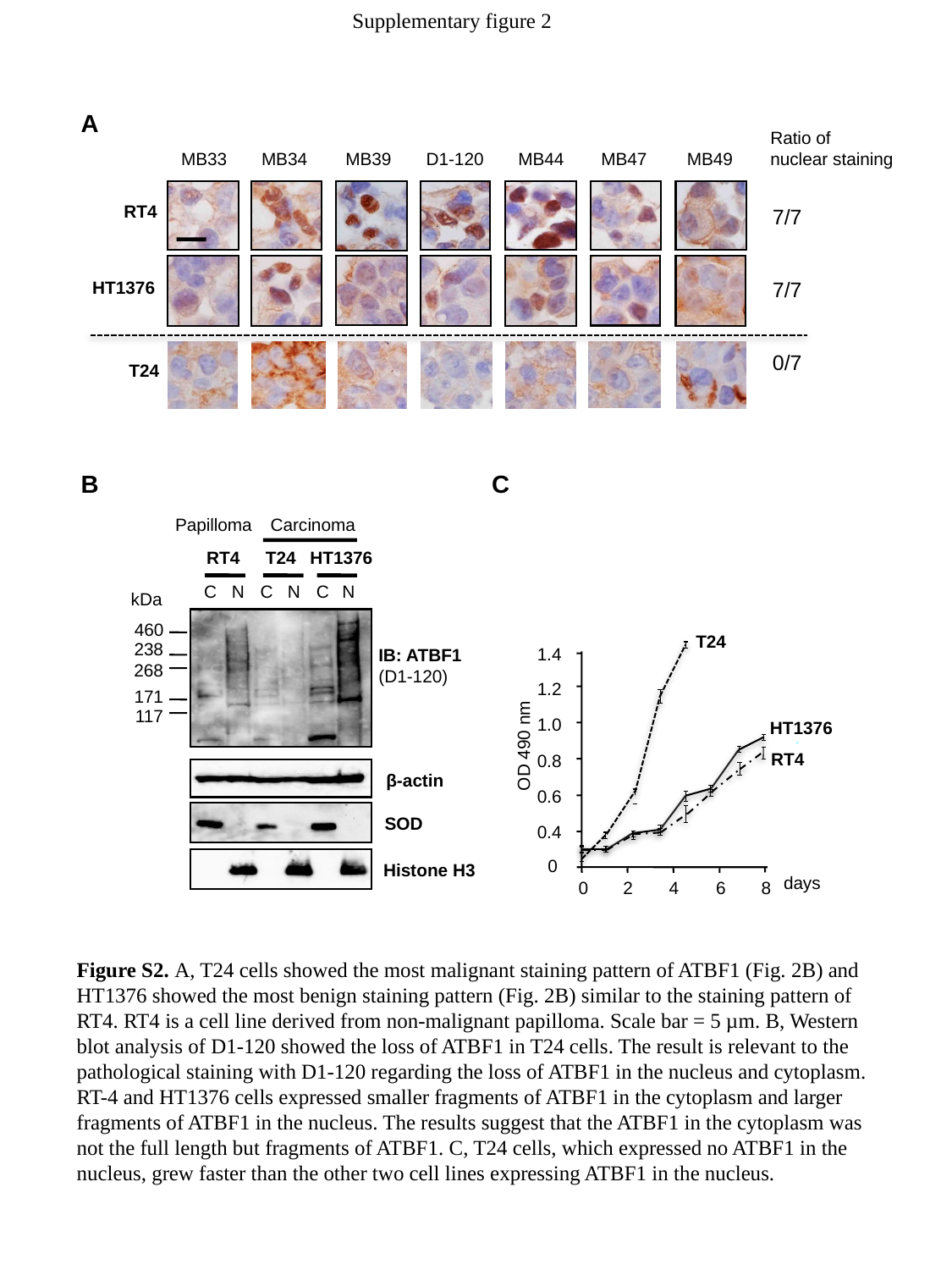

Supplementary figure 2
A
Ratio of
nuclear staining
MB33
MB34
MB39
D1-120
MB44
MB47
MB49
RT4
7/7
HT1376
7/7
0/7
T24
B
C
Papilloma
Carcinoma
RT4
T24
HT1376
C
N
C
N
C
N
kDa
460
T24
238
IB: ATBF1
(D1-120)
1.4
268
1.2
171
117
HT1376
1.0
OD 490 nm
RT4
0.8
β-actin
0.6
SOD
0.4
Histone H3
0
days
0
2
4
6
8
Figure S2. A, T24 cells showed the most malignant staining pattern of ATBF1 (Fig. 2B) and HT1376 showed the most benign staining pattern (Fig. 2B) similar to the staining pattern of RT4. RT4 is a cell line derived from non-malignant papilloma. Scale bar = 5 µm. B, Western blot analysis of D1-120 showed the loss of ATBF1 in T24 cells. The result is relevant to the pathological staining with D1-120 regarding the loss of ATBF1 in the nucleus and cytoplasm. RT-4 and HT1376 cells expressed smaller fragments of ATBF1 in the cytoplasm and larger fragments of ATBF1 in the nucleus. The results suggest that the ATBF1 in the cytoplasm was not the full length but fragments of ATBF1. C, T24 cells, which expressed no ATBF1 in the nucleus, grew faster than the other two cell lines expressing ATBF1 in the nucleus.
