# Supplementary material for: A diagnostic marker for superficial urothelial bladder carcinoma: lack of nuclear ATBF1 (ZFHX3) by immunohistochemistry suggests malignant progression
Source: BMC Cancer. 2016 Oct 18;16:805. doi: 10.1186/s12885-016-2845-5 (PMC5070376; doi:10.1186/s12885-016-2845-5)
Supplement: Additional file 4: Figure S4. — ATBF1 is highly expressed in cells at the epithelial zone associated with the expression of E-cadherin. A, Pathological specimens of bladder carcinoma were stained by hematoxylin and eosin, E-cadherin, and anti-ATBF1 antibodies (MB33, MB34, MB39, D10120, MB44, MB47 and MB49). Bladder lumen (L), epithelial zone (E), and mesenchymal zone (M) are shown. A transition from higher expression of ATBF1 in the E zone to lower expression of ATBF1 in the M zone was observed. The basement membrane is not clear between the E and M zones because of invasive cancer. Scale bar = 5 μm. B, Schematic explanation of the function of ATBF1 in inducing E-cadherin. ATBF1 interacts with protein inhibitor of activated STAT3 (PIAS3) to suppress STAT3 signaling [21]. Activation of STAT3 should activate the zinc transporter LIV1 and introduce zinc ions into the cells. The zinc ion is a regulatory factor for Snail to suppress E-cadherin. Therefore, zinc transporters play an important role in the migration of cells during embryogenesis [20]. Fragments of ATBF1 may have a dominant negative effect on suppressing STAT3, whereas they promote STAT3 signaling and suppress the expression of E-cadherin, which might be the basis of invasion and metastasis. (PPTX 1682 kb) [file 12885_2016_2845_MOESM4_ESM.pptx]

## Slide 1
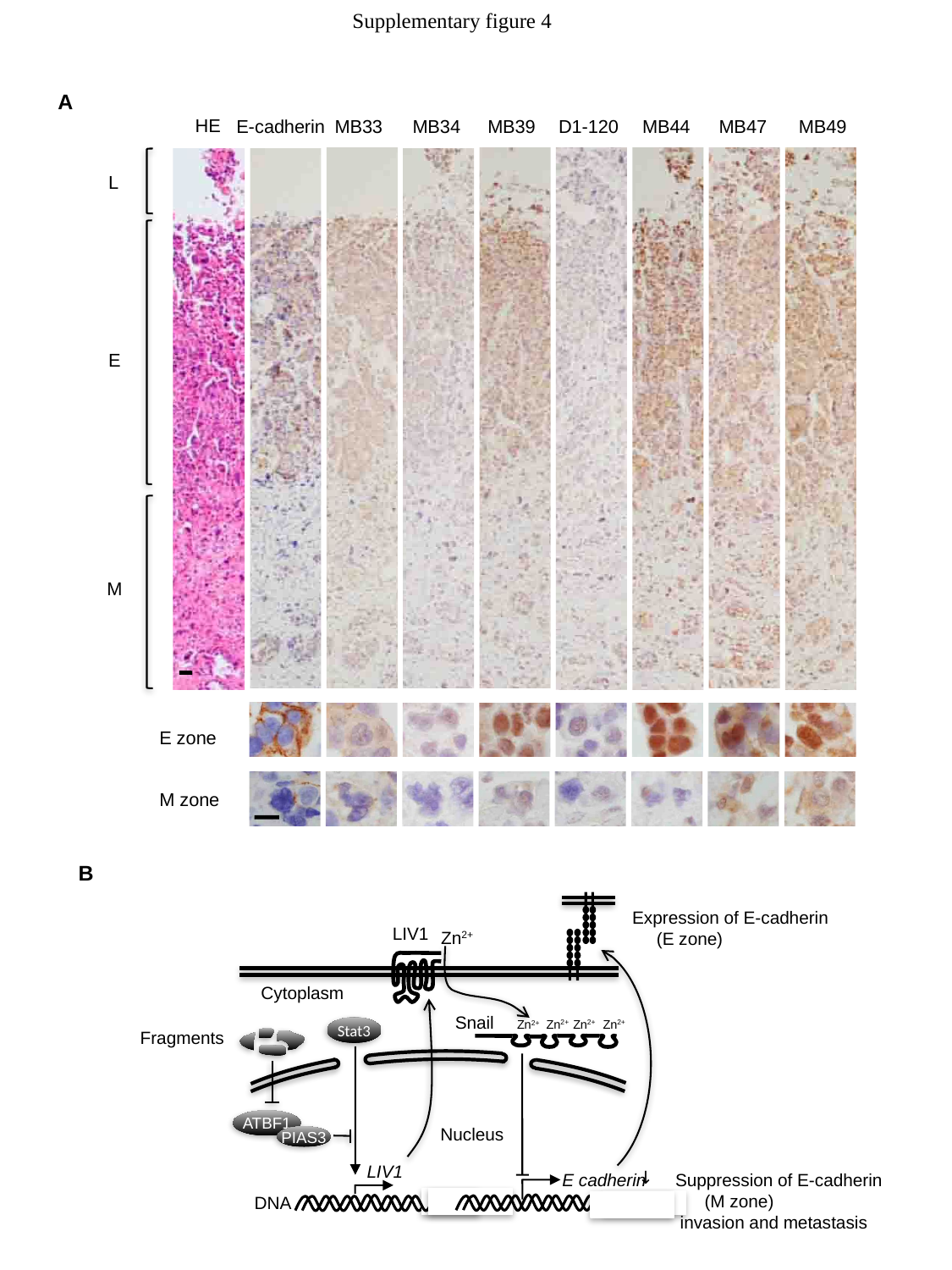

Supplementary figure 4
A
HE
E-cadherin
MB33
MB34
MB39
D1-120
MB44
MB47
MB49
L
E
M
E zone
M zone
B
Expression of E-cadherin
 (E zone)
LIV1
Zn2+
Cytoplasm
Snail
Zn2+
Zn2+
Zn2+
Zn2+
Stat3
Fragments
ATBF1
Nucleus
PIAS3
LIV1
↓
 E cadherin
Suppression of E-cadherin
 (M zone)
 invasion and metastasis
DNA

## Slide 2
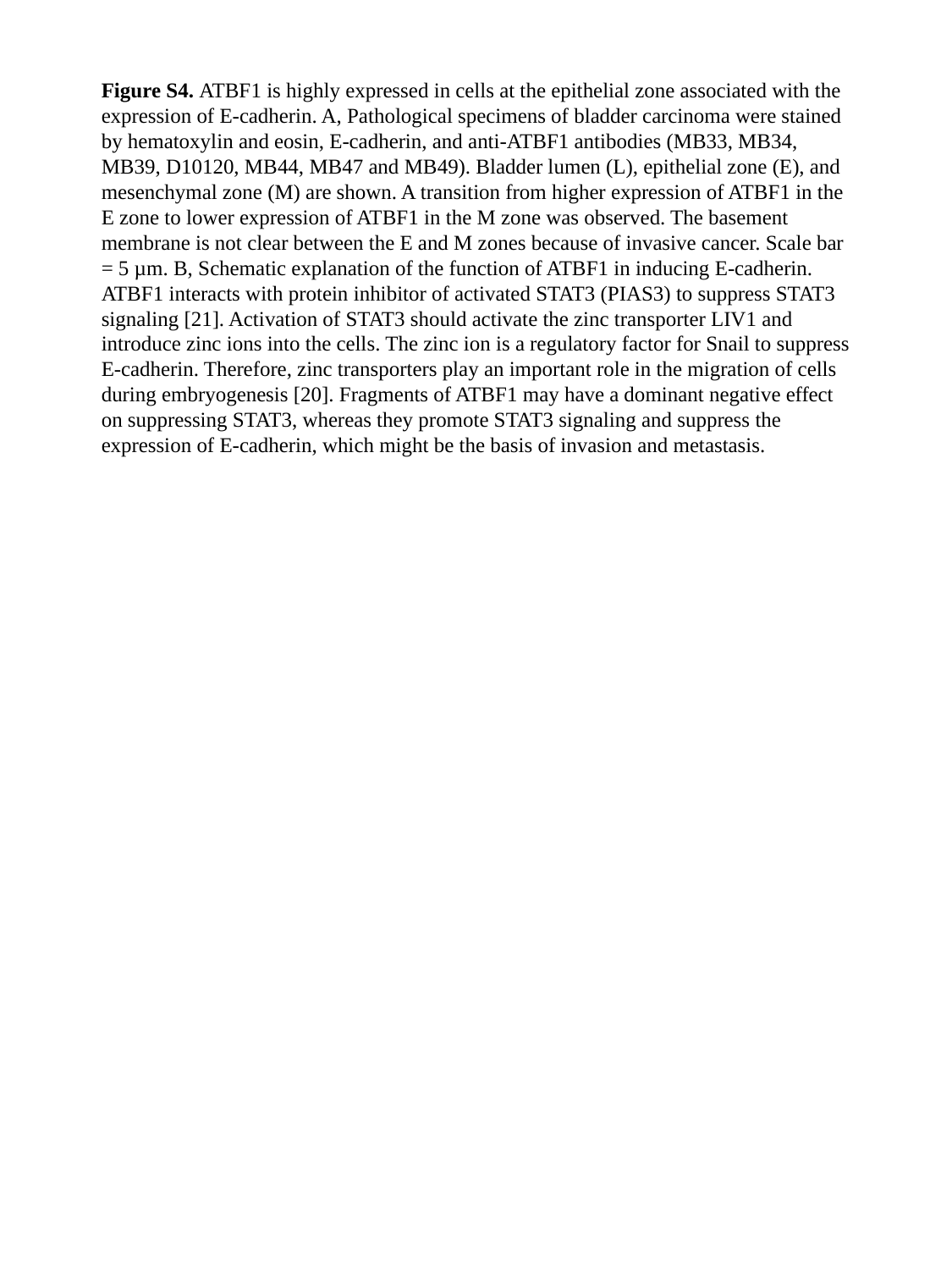

Figure S4. ATBF1 is highly expressed in cells at the epithelial zone associated with the expression of E-cadherin. A, Pathological specimens of bladder carcinoma were stained by hematoxylin and eosin, E-cadherin, and anti-ATBF1 antibodies (MB33, MB34, MB39, D10120, MB44, MB47 and MB49). Bladder lumen (L), epithelial zone (E), and mesenchymal zone (M) are shown. A transition from higher expression of ATBF1 in the E zone to lower expression of ATBF1 in the M zone was observed. The basement membrane is not clear between the E and M zones because of invasive cancer. Scale bar = 5 µm. B, Schematic explanation of the function of ATBF1 in inducing E-cadherin. ATBF1 interacts with protein inhibitor of activated STAT3 (PIAS3) to suppress STAT3 signaling [21]. Activation of STAT3 should activate the zinc transporter LIV1 and introduce zinc ions into the cells. The zinc ion is a regulatory factor for Snail to suppress E-cadherin. Therefore, zinc transporters play an important role in the migration of cells during embryogenesis [20]. Fragments of ATBF1 may have a dominant negative effect on suppressing STAT3, whereas they promote STAT3 signaling and suppress the expression of E-cadherin, which might be the basis of invasion and metastasis.

## Slide 3
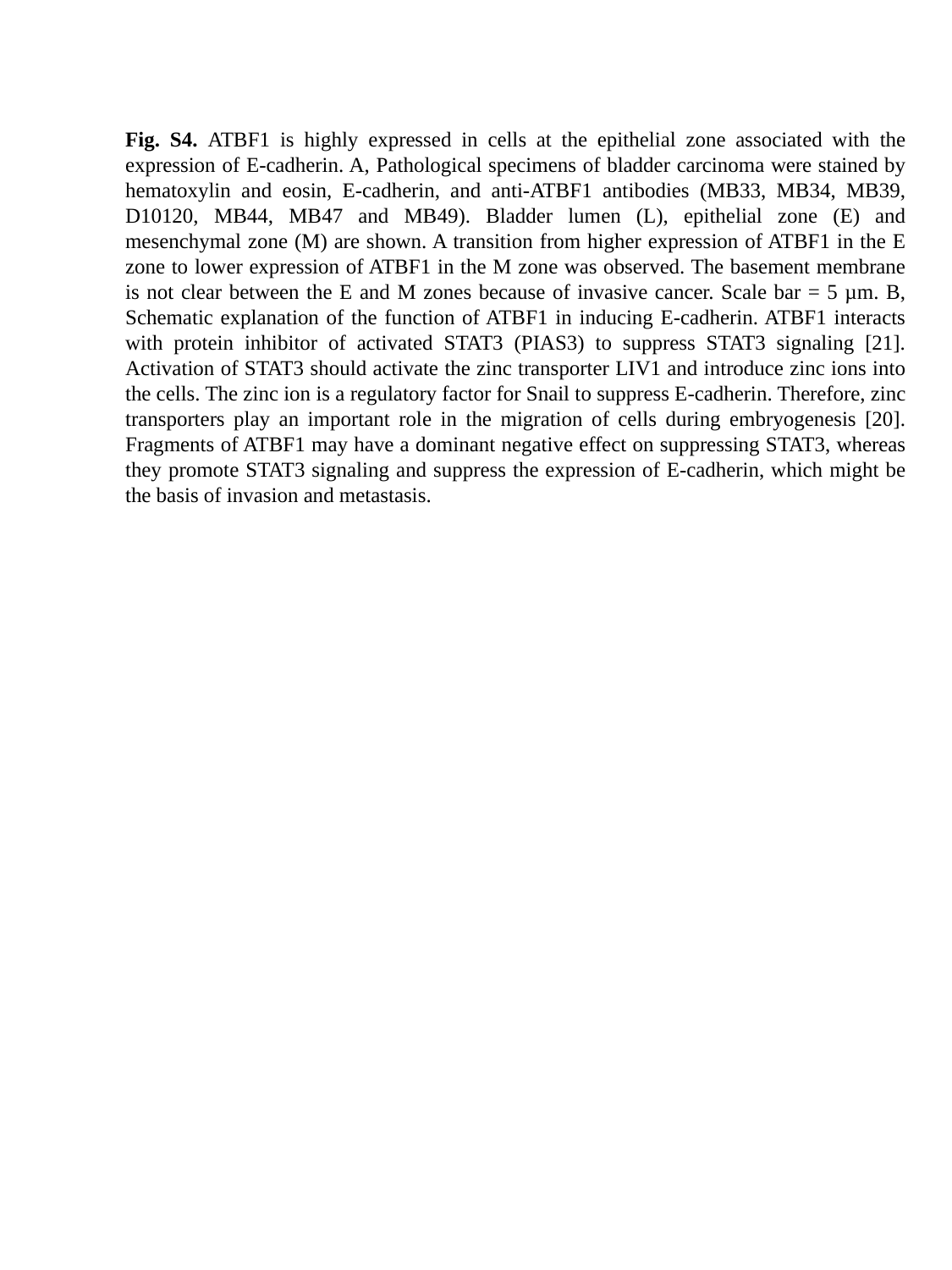

Fig. S4. ATBF1 is highly expressed in cells at the epithelial zone associated with the expression of E-cadherin. A, Pathological specimens of bladder carcinoma were stained by hematoxylin and eosin, E-cadherin, and anti-ATBF1 antibodies (MB33, MB34, MB39, D10120, MB44, MB47 and MB49). Bladder lumen (L), epithelial zone (E) and mesenchymal zone (M) are shown. A transition from higher expression of ATBF1 in the E zone to lower expression of ATBF1 in the M zone was observed. The basement membrane is not clear between the E and M zones because of invasive cancer. Scale bar = 5 µm. B, Schematic explanation of the function of ATBF1 in inducing E-cadherin. ATBF1 interacts with protein inhibitor of activated STAT3 (PIAS3) to suppress STAT3 signaling [21]. Activation of STAT3 should activate the zinc transporter LIV1 and introduce zinc ions into the cells. The zinc ion is a regulatory factor for Snail to suppress E-cadherin. Therefore, zinc transporters play an important role in the migration of cells during embryogenesis [20]. Fragments of ATBF1 may have a dominant negative effect on suppressing STAT3, whereas they promote STAT3 signaling and suppress the expression of E-cadherin, which might be the basis of invasion and metastasis.
